# Experimental evidence on post-program effects and spillovers from an agriculture-nutrition program

**DOI:** 10.1016/j.ehb.2019.100820

**Published:** 2020-01

**Authors:** Andrew Dillon, Lilia Bliznashka, Deanna Olney

**Affiliations:** a Northwestern University, 2211 CAMPUS DRIVE, Evanston, 60208, United States; b Harvard University, 665 Huntington Ave Building I, Floor 11 Boston MA, USA 02115; c International Food Policy Research Institute, 1201 I St, NW, Washington, DC 20005

**Keywords:** Childhood nutrition, Agricultural production, Spillover effects, Randomized control trials, Burkina Faso

## Abstract

Integrated agricultural-nutrition programs are often implemented under the premise that program effects are durable and spillover. This paper estimates one year post-program effects, three-year aggregate program effects and spillover effects using treated and untreated household cohorts. Two treatment interventions implemented agricultural interventions with behavior change communication strategies varying implementers using either village health committees or older female leaders. In the post-program period, program effects deteriorated relative to program period impacts documented in Olney et al. (2015), but the three-year agricultural, nutrition knowledge, health care practices and severe anemia impacts remained statistically significant. Despite the non-rival nature of nutrition education and promoted production techniques, there is little evidence of agricultural technology or health knowledge spillovers to non-treated households within treatment communities. Spillover effects measured for appropriate treatment of diarrhea (10 pp increase in giving rehydration salts rather than traditional medicine), wasting (20 pp lower probability of wasting) and children’s anemia status (7 pp reduction in severe anemia) significantly improve in later cohorts. The aggregate program effects and spillovers are generally robust to multiple hypothesis testing.

## Highlights

• An earlier agriculture-nutrition program prevented wasting and anemia in children.
• Program impacts were due to positive shifts in nutrition knowledge and practices.
• There was limited evidence of knowledge diffusion to non-treated households.
• Feeding practices and anthropometric measures did improve in later cohorts.

## 1 Introduction

Post-program and spillover effects are often intended consequences of agriculture and nutrition programs designed to increase their potential effect over time (World Bank, 2007). Programs may have spillover effects if they generate non-rival goods which diffuse in communities or, to the contrary, lead to disadoption after the program implementation period because monitoring, knowledge decay or the return on the productive or nutritional investment is no longer positive. The technology adoption literature provides many examples of the diffusion of new technologies (Munshi 2004, Bandiera and Rasul 2006, Beaman et al., 2015), the diffusion of knowledge about new technologies (Beaman and Dillon, 2018) and the disadoption after the initial period take-up (Moser and Barrett, 2006, Duflo et al., 2011, Gilligan et al., 2014). The behavior change communication (BCC) literature in nutrition also documents increased knowledge, yet low adoption of improved infant and young child feeding (IYCF) practices, in response to nutrition education campaigns (World Health Organization, 2010). Estimation of post-program and spillover effects due to nutrition interventions is limited in the developing country context (Benjamin-Chung et al., 2017), though in developing countries health spillovers between siblings (Ho, 2017) and from health education programs (Mora et al., 2015) have been documented. This paper builds on these two literatures by, first, testing whether program impacts endure in the first year after program implementation ends among treated households, and second to test for spillover effects by estimating whether implementation period agricultural and nutritional program impacts estimated in Olney et al. (2015) are also measured in non-treated households in treatment villages. We disentangle the mechanisms of nutritional post-program effects and spillovers by estimating treatment effects on nutritional ‘inputs’ such as mothers’ health and nutrition knowledge and agricultural production mechanisms independently.

To estimate spillovers of agricultural production techniques, health knowledge, and children’s nutritional status, we build on a cluster-randomized control trial conducted in partnership with Helen Keller International (HKI) in Burkina Faso. From 2010–2012, HKI implemented an Enhanced Homestead Food Production (EHFP) program in Burkina Faso with the specific objectives of improving women’s agricultural production of nutrient-rich foods and children’s nutritional status.^1^ Two treatment groups received a standardized production intervention and a nutrition behavior change communication (BCC) intervention with the only difference between the two treatment groups being the BCC implementers: either older women leaders (OWL) or health committee (HC) members. The production interventions were standardized across each treatment group focused on knowledge of the nutritional content of specific foods that were locally grown to encourage adoption, access to inputs, including seed and tools, to establish village gardens, and materials to encourage the establishment of a homestead garden. It was expected that the program would have the biggest impact on improving the nutritional status of children during the first 1,000 days of life, therefore households with children 3–12 months of age were targeted by the program. Mothers with children 3–12 months of age living in the treatment villages were invited to participate in the EHFP program, along with their husbands and children. Our previous analysis of the program impacts (Olney et al., 2015) was based on the analysis of a baseline study that was conducted between February and May 2010 and an endline study that was conducted between February and June 2012. In 2012, during the endline study, an additional cohort of younger children 3–12 months old were sampled in treatment and control villages. A post-treatment follow-up survey was also administered between March and June 2013 to track this younger cohort of children, their mothers and households, in addition to the original households interviewed in treatment and control villages at baseline.

The EHFP program was successful in improving women’s agricultural production during the program implementation period, specifically women’s production of vitamin A-rich foods. No additional agricultural treatment effects on treated households or non-treated households were found after the program implementation period. Women’s use of manure and fertilizer decreased in the post-program period, though manure use and number of plots cultivated by women were still statistically significantly higher over the three-year period of the study in treatment as compared to control villages. Increases in women’s production of vitamin A-rich crops found during the program implementation, however, were not sustained in the post-program period. These results suggest that program extension advice, monitoring and input provision were critical to the small production gains found during the program implementation period. No spillover effects on new cohort households were found in terms of utilization of inputs or production outcomes.

We also found positive program effects on mothers’ knowledge of optimal IYCF and hygiene and healthcare practices as compared to non-treated mothers. The analysis of the post-program period impacts suggests that mothers’ health and nutrition knowledge deteriorated after the end of the program, though knowledge gains were statistically significantly higher among treated as compared to non-treated mothers for the over the three-year period of the study. Spillovers on health care practice knowledge and exclusive breastfeeding practices for children less than six months of age were estimated between treatment village cohorts. We estimate a 23 percentage point increase in later OWL treatment village cohorts on exclusive breastfeeding of children less than 6 months old. In the HC and OWL later treatment cohorts, there was a 10–11 percentage point decrease in treating diarrhea with traditional medicine and in the HC treatment cohorts, a corresponding 10 percentage point increase in giving rehydration salts for diarrhea treatment.

With respect to children’s nutritional outcomes, the primary findings during the program implementation period were that the EHFP program improved hemoglobin concentration and decreased the prevalence of anemia, wasting and diarrhea among children living in HC villages relative to those living in control villages (Olney et al., 2015). In the post-implementation period, no further increases in hemoglobin concentration were found. However, the relative increase in hemoglobin concentration that was found among children 3–5.9 months of age at baseline were sustained one-year after the program ended, although the impact estimate for the three-year study period was only marginally statistically significant. No spillover effects were measured on the new cohort of children 3–12 months of age at endline in the post-implementation period in terms of their anemia status or hemoglobin level. During the program (2010–2012), the prevalence of wasting among children 3–12 months of age at baseline living in the HC villages relative to those living in the control villages decreased significantly by 8.4 percentage points. In the three-year estimates, the prevalence of wasting in HC villages had declined by 5.4 percentage points more as compared to control villages, but this difference was not statistically significant. In addition, there is some evidence that there were significant protective effects of the program on preventing wasting among children born into treated households during the program period. In control villages, the prevalence of wasting increased from 2012 to 2013 among children 3–12 months of age. In 2012, there was no change in wasting in OWL villages, and in HC villages there was a decline, resulting in 19.5 and 20.4 percentage point differences between each of the treatment groups and the control group in the prevalence of wasting from 2012 to 2013. These differences were statistically significant and indicate a potential role of the EHFP program in protecting children’s nutritional status during this vulnerable age.

Because there are multiple hypotheses for agricultural, knowledge, practices, or nutritional set of measures, we correct all estimates using multiple hypothesis testing following Anderson (2008) to assess the robustness of our results. Among the statistically significant effects estimated for the post-program period, three year overall program effects or spillover effects, we report corrected p-values that account for multiple hypothesis testing. The overall program effects are robust to multiple hypothesis testing (9 out of 11 statistically significant results), while only half the post-program effects are statistically significant after multiple hypothesis corrections (3 out of 6 results). The spillover effects are generally robust to multiple hypothesis corrections (4 out of 6 results).

In the next section of the paper, we present the experimental design, while the third section details the post-program and spillover effects identification strategy. The fourth section presents the results, while the last section concludes.

## 2 Experimental design

### 2.1 Program description

HKI’s EHFP program implemented in the province of Gourma in Burkina Faso consisted of an agricultural production component and a nutrition and health BCC strategy. The agricultural production activities included input distribution (e.g. seeds, saplings, chicks and small gardening tools) and agricultural training provided by female village farm leaders at village model farms. Program beneficiaries started their own homestead food production activities after the receipt of inputs and training. Production activities primarily included the promotion of micronutrient-rich fruits and vegetables, eggs and poultry production. The BCC strategy was designed using the essential nutrition actions framework that focuses on seven practices: women’s nutrition, anemia prevention and control (e.g. intake of iron-rich foods and use of bednets to prevent malaria), iodine intake, prevention of vitamin A deficiency, breastfeeding practices, complementary feeding practices, and nutritional care for sick and severely malnourished children (Guyon et al., 2009). Beneficiary women received bimonthly home visits from either an older woman leader (OWL) or a health committee (HC) member who implemented through these visits a behavior change communication curriculum during which beneficiaries were taught about optimal health and nutrition practices, and discussed successes and challenges related to the adoption of these practices.

### 2.2 Study design and participants

The EHFP program was evaluated using a cluster-randomized controlled trial^2^ to estimate the effects of the program on agricultural production, knowledge, practices and ultimately nutritional outcomes of young children^3^ . Villages in four departments in the Gourma province (Diapangou, Diabo, Tibga, Yamba) were selected for inclusion if they had access to water in the dry season to enable participation in the agricultural intervention. Fifty-five villages were stratified by department and village size and randomized into three groups: 1) control group which received no interventions (25 control villages); 2) OWL group that received the agricultural production intervention with the BCC strategy implemented by older women leaders (OWL) (15 OWL villages); and 3) HC group that received the same agricultural production intervention with the BCC strategy implemented by health committee (HC) members (15 HC villages). The two types of behavior change implementers were selected because the effectiveness in improving knowledge and eliciting behavior change may vary by the type of implementer delivering the messages. In rural areas, HC members delivered health and nutrition interventions and could facilitate direct links with health services, while OWLs were the main providers of ante- and post-natal counseling and delivery care and thus could be more influential in changing infant and young child feeding and care practices.

Within the selected villages (n = 55) all women with children 3–12 months of age were invited to participate in the study.^4^ Trained fieldworkers explained the study to eligible households and informed consent was obtained from either the household head or the mother of the selected child. We used a longitudinal design and followed the same households, mothers and children over the two-year program implementation period. The baseline study was conducted between February and May of 2010 (when children were 3–12 months of age), and the endline survey between February and June of 2012 (when children were 24–40 months of age).

In 2012, during the endline study, an additional cohort of younger children 3 to 12 months of age was sampled in treatment and control villages from both treated households, who participated in the EHFP program or impact evaluation, and from a new cohort of non-treated households in both treatment and control villages. A post-treatment follow-up survey was administered between March and June 2013 to track this younger cohort of non-treated children, and their households, in addition to the original treated households, mothers and children interviewed in treatment and control villages at baseline and endline.

Thus, we have a cohort of treated households, mothers and children 3–12 months of age at baseline interviewed at baseline in 2010, endline in 2012 and follow-up in 2013. In addition, we have a cohort of younger non-treated children 3–12 months of age at endline born into the treated and non-treated households in treatment and control villages. This younger cohort and their households were surveyed in 2012 and again in 2013. Half of these younger cohort children are siblings with the same mother, and the remaining are siblings with a different mother (co-wife) and cousins due to the frequency of polygamous household structures. All age eligible children were interviewed in both the original and follow-up cohorts.

## 3 Post-program, three year effects, and spillover effect identification

Three treatment effects are specified to measure different types of program effects after the program implementation period (2010–2012): a post-program effect (2012–2013), a three-year effect of the program (2010–2013) and spillover effects to non-beneficiary households. The specifications are estimated with the following regressions for the 2010 cohort restricting the sample to treated households in OWL and HC villages and control households from the 2010 cohort:

Program implementation effect:

(1) $$\Delta H_{2012}-H_{2010}=\;\beta^{HC}{HC}_{2010}+\;\beta^{OWL}{OWL}_{2010}+\;\gamma X_{2010}+\;\varepsilon$$

Post-program effect:

(2) $$\Delta H_{2013}-H_{2012}=\;\beta^{HC}{HC}_{2010}+\;\beta^{OWL}{OWL}_{2010}+\;\gamma X_{2010}+\;\varepsilon$$

Three-year effect:

(3) $$\Delta H_{2013}-H_{2010}=\;\beta^{HC}{HC}_{2010}+\;\beta^{OWL}{OWL}_{2010}+\;\gamma X_{2010}+\;\varepsilon$$

where $H_{Year}$ is the change in program indicator variable between survey rounds which could be either a household-, mother- or child-level indicator. HC indicates treatment in the HC group (1 if household is in a HC village, 0 if not) and OWL indicates treatment in the OWL group (1 if household is in an OWL village, 0 if not) using the treatment assignment at baseline (child between 3–12 months old in 2010). The specification also includes baseline characteristics of the mother or child depending on the program indicator variable chosen. We report the program implementation effects in the appendix of the paper as reference for the interpretation of the post-program and three year effects.

The identification of spillover effects in our study builds on the recent literature (Baird et al., 2018; Angelucci and Giacomo De, 2009; Miguel and Kremer, 2004)) which uses the exogenous variation in program assignment and eligibility status. As households were screened for treatment eligibility if households had a child between 3–12 months at baseline and the new cohort selected in 2012 used the same survey selection criterion, the group on whom spillovers would occur is well defined in our data.

To estimate potential program spillovers the following specifications are estimated for the 2012 cohort which includes non-beneficiary households with children 3–12 months of age at the time of the endline survey in 2012 in treatment and control villages. This is a post-program spillover effect on the subsequent cohort of households with children who would have been targeted by the intervention had they been born earlier.

(4) $$\Delta H_{2013}-H_{2012}=\;\beta^{HC}{HC}_{2012}+\;\beta^{OWL}{OWL}_{2012}+\;\gamma X_{2012}+\;\varepsilon$$

where $H_{Year}$ is the change in program indicator variable between survey rounds which could be either a household-, mother- or child-level indicator. HC indicates treatment in the HC group (1 if household is in a HC village, 0 if not) and OWL indicates treatment in the OWL group (1 if household is in an OWL village, 0 if not) restricting the sample to households who had a child between 3–12 months old in 2012. The specification also includes characteristics of the mother or child depending on the program indicator variable chosen.

As an alternative spillover measure we also estimate the within village cohort spillover effect:

(5) $$H_{cohort}=\;\beta^{HC}{HC}_{cohort}+\;\beta^{OWL}O{WL}_{cohort}+\;\gamma Year+\;\gamma X_{cohort}+\varepsilon$$

where $H_{cohort}$ is children’s nutritional status from the cohorts selected from the baseline in 2010 or new households in 2012 from treatment villages^5^ . A control variable for the cohort year, Year, and observable characteristics in the cohort year, $X_{cohort}$, are also included in the specification. The HC and OWL coefficients represent the mean difference between the old and new cohort households in the HC or OWL villages. The year fixed effect addresses differences between cohorts as inter-annual variation due to agricultural production and disease environment variation are likely. The standard errors (SE) of the above regressions are all corrected for clustering at the village level, the unit at which treatment was assigned.

A strength of the above econometric strategy is the direct testing of production, knowledge, practice and nutrition outcomes which the program was designed to impact. One statistical concern is that because there are multiple primary and secondary effects of an integrated agriculture-nutrition intervention, our results could over report false positive effects without corrections for multiple hypothesis testing. To address this concern, we first present all treatment effect results. We follow Anderson (2008) to correct treatment effect p-values within agricultural, knowledge, practices and nutritional outcome groupings among related outcome measures that are statistically significant to assess their robustness to multiple-inference corrections. The paper’s main results and their robustness with Anderson’s q-values are presented in Table 5.

Attrition between the panel cohorts are also a potential source of bias to treatment effect estimation. We analyzed this potential source of bias in Olney et al. (2015) and Dillon et al. (2019). In the treatment villages, 13.9 percent of households attrited, with no statistically significant difference between the two treatment groups. In the control villages, 19.2 percent of control group households attrited. This difference resulted in a statistically significant *t*-test of the difference in mean attrition at the 1% level of statistical significance. Female headed households, households with male household members and fewer children were more likely to attrit from the household panel, but predictors such as education, durable asset values, land size or baseline production were not significant determinants of the attrition rate. We address differences in attrition between the treatment and control groups by first analyzing whether these differences leads to observable characteristic differences in balancing tests of the sample at baseline in Table A1 in Supplementary material. We also estimate attrition weights^6^ using inverse probability weighting and use these weights in treatment effect estimation.

## 4 Results

### 4.1 Balancing tests and descriptive statistics

To test whether the sample has statistical balance in observable characteristics between the treatment and control groups, balancing tests on household- and child-level characteristics are estimated for the 2010 and 2012 cohorts in Tables A1 and A2 in Supplementary material, respectively. We report household characteristics by treatment group, HC or OWL, and control, as well as the p-value of the test of mean equality between the three groups for the non-attrited sample. Household characteristics such as household size, value of men’s and women’s assets, and household head’s and mother’s educational status were all balanced across the three groups at initial randomization in 2010. One housing characteristic, having a dirt floor, was not balanced between the treatment and the control groups, with higher prevalence of dirt flooring in HC and OWL villages, indicating a lower level of housing quality. Children’s characteristics, including the gender and age, were balanced at baseline, though children’s nutritional indicators were not. Household attrition does not seem to be a significant source of observable characteristic imbalance, though there are differences in attrition rates between the treatment and control groups.

In Table A2 in Supplementary material, we test the mean equality of the same household and child characteristics at selection for the 2012 cohort^7^ . Though villages were not re-randomized in 2012 as the objective of collecting information was to estimate spillover effects, we compare mean characteristics across the treatment and control groups to assess whether treatment status potentially affected household composition and welfare characteristics, apart from the nutritional, production or knowledge indicators on which we could potentially observe a spillover. Table A2 in Supplementary material reports that household characteristics, value of assets and livestock owned, as well as children’s characteristics were well balanced across the treatment and control groups. Children’s nutritional indicators were not all balanced across groups, particularly children’s hemoglobin status.

We test for balance between the old and new cohorts and report those results in the Table A3 in Supplementary material. Due to variation in agricultural production and disease environment between years, it is not necessarily expected that cohort characteristics will be balanced between years. The balancing tests demonstrate that household and child nutritional indicators are not balanced between the cohorts while characteristics such as child age, child gender, household size and women’s assets are balanced between cohorts. Differences between cohorts motivate the inclusion of time fixed effects in the between cohort spillover specification described above.

### 4.2 Post-program, three-year program, and spillover effects

HKI’s EHFP program in Burkina Faso was designed to improve household agricultural production of nutrient-rich foods and the nutritional status of the targeted children who were 3–12 months of age at the time of the baseline study through an integrated set of production and nutrition interventions. We estimate treatment effects of the program on the primary outcomes – household agricultural production of nutrient-rich foods, women’s health and nutrition-related knowledge, IYCF practices, and children’s nutritional status including anemia and anthropometric measures.

As expected, HKI’s EHFP program was successful in increasing women’s agricultural production during the program implementation period, particularly women’s production of vitamin A-rich foods (Table A4 in Supplementary material). Although there was no overall impact on household or on men’s production, impacts on women’s agricultural production were fairly consistent, albeit small, across the different types of crops. These results are consistent with other studies that have shown small impacts of small-scale agriculture interventions on household food production (World Bank, 2007; Masset et al., 2012; Olney et al., 2009; Dillon et al., 2019).

In the post-program period, women’s use of manure and fertilizer decreased in the post-implementation period, though manure use and the number of plots cultivated by women were still statistically higher over the three-year period of the study. Increases in women’s production of vitamin A-rich foods found during the program implementation period were not sustained in the post-implementation period (Table 1-Panel A). While the number of plots cultivated by women and manure use were statistically higher in the treatment groups relative to the control over the 2010–2013 period, one-year post-program implementation agricultural production effects were not sustained (Table 1-Panel B). These results suggest that program extension advice, monitoring and input provision were critical to the small production gains found during the program implementation period. No spillover effects on non-beneficiary households were measured (Table 1-Panel C), suggesting that if agricultural programs are expected to improve children’s nutritional status through increased food availability, the scale of the program and post-program extension advice are likely important program design features.

**Table 1:** Production Effects of Agricultural Interventions.

Panel A - Post-program impact on beneficiary households, 2012-2013										
*Inputs*	Hectares cultivated - men	Hectares cultivated -women	Number of plots -men	Number of plots-women	Fertilizer use-men (%)	Fertilizer use-women (%)	Pesticide/ insecticide/herbicide use-men (%)	Pesticide/ insecticide/herbicide use-women (%)	Manure use-men (%)	Manure use-women (%)
Treatment	−0.37	0.31	−0.14	−0.83	0.38	−5.29*	−5.10	−0.94	−0.68	−26.86***
	(0.31)	(0.39)	(0.32)	(0.55)	(6.68)	(2.71)	(5.40)	(2.53)	(7.79)	(4.71)
Mean at baseline – control group	2.68 (4.06)	0.67 (1.73)	3.30 (1.99)	4.01 (2.98)	25.61 (43.68)	14.18 (34.91)	7.95 (27.08)	3.55 (18.52)	49.90 (50.04)	53.49 (49.92)

Number of obs. (N)	1,157	1,157	1,157	1,157	1,157	1,157	1,157	1,157	1,157	1,157
p-value	0.24	0.44	0.65	0.13	0.95	0.06	0.35	0.71	0.93	0.00
*Production*	Total production - men (kg)	Total production -women (kg)	Grains and tubers -men (kg)	Grains and tubers-women (kg)	Legumes, nuts and pulses-men (kg)	Legumes, nuts and pulses-women (kg)	Other fruits and vegetables-men (kg)	Other fruits and vegetables-women (kg)	Vitamin A-rich crops-men (kg)	Vitamin A-rich crops-women (kg)
Treatment	241.63	12.11	124.20	48.27	−23.41	27.59	2.15	2.50	−1.16	−1.08
	(300.72)	(147.12)	(162.74)	(72.85)	(42.81)	(28.92)	(22.49)	(7.62)	(25.85)	(2.70)
Mean at baseline – control group	1,992.8 (2676.1)	336.8 (1009.3)	1140.4 (2165.1)	125.49 (518.57)	73.73 (315.13)	43.67 (114.77)	9.28 (108.11)	12.17 (70.53)	23.37 (527.82)	3.87 (55.60)
Number of obs. (N)	1,157	1,157	1,157	1,157	1,157	1,157	1,157	1,157	1,157	1,157
p-value	0.43	0.93	0.45	0.51	0.59	0.34	0.92	0.74	0.96	0.69

Panel B - Overall impact on beneficiary households, 2010-2013										
*Inputs*	Hectares cultivate -men	Hectares cultivated-women	Number of plots-men	Number of plots-women	Fertilizer use-men (%)	Fertilizer use-women (%)	Pesticide/ insecticide/herbicide use-men (%)	Pesticide/ insecticide/herbicide use-women (%)	Manure use-men (%)	Manure use-women (%)
Treatment	0.03	−0.25	0.16	1.40**	−2.83	3.40	0.56	1.66	6.52	20.72***
	(0.37)	(0.20)	(0.27)	(0.56)	(6.41)	(3.30)	(4.03)	(2.54)	(7.53)	(5.08)
Mean at baseline – control group	3.01 (2.73)	0.98 (4.72)	3.50 (1.99)	2.36 (2.48)	23.83 (42.63)	4.18 (20.03)	8.29 (27.60)	1.27 (11.21)	53.19 (49.93)	9.16 (28.87)

Number of obs. (N)	1,156	1,156	1,156	1,156	1,156	1,156	1,156	1,156	1,156	1,156
p-value	0.94	0.21	0.56	0.02	0.66	0.31	0.89	0.51	0.39	0.00
*Production*	Total production- men (kg)	Total production-women (kg)	Grains and tubers-men (kg)	Grains and tubers-women (kg)	Legumes, nuts and pulses-men (kg)	Legumes, nuts and pulses-women (kg)	Other fruits and vegetables-men (kg)	Other fruits and vegetables-women (kg)	Vitamin A-rich crops-men (kg)	Vitamin A-rich crops-women (kg)
Treatment	125.08	47.09	151.24	76.90	−30.74	6.67	−17.12	3.34	13.45	2.54
	(302.82)	(131.23)	(144.49)	(70.10)	(27.91)	(20.11)	(18.22)	(3.03)	(18.29)	(1.60)
Mean at baseline – control group	604.00 (647.00)	108.00 (248.00)	391.00 (573.00)	38.00 (157.00)	23.07 (69.00)	21.91 (99.00)	4.70 (57.00)	1.74 (16.00)	8.51 (139.00)	0.18 (4.40)

Number of obs. (N)	1,156	1,156	1,156	1,156	1,156	1,156	1,156	1,156	1,156	1,156
p-value	0.68	0.72	0.30	0.28	0.28	0.74	0.35	0.27	0.47	0.12

Panel C - Post-program impact on new cohort households, 2012-2013										
*Inputs*	Hectares cultivated-men	Hectares cultivated-women	Number of plots-men	Number of plots-women	Fertilizer use-men (%)	Fertilizer use-women (%)	Pesticide/ insecticide/herbicide use-men (%)	Pesticide/ insecticide/herbicide use-women (%)	Manure use-men (%)	Manure use-women (%)
Treatment	0.25	−0.10	−0.16	0.03	−8.14	−0.65	−5.46	−3.14	−5.92	4.79
	(0.24)	(0.11)	(0.27)	(0.40)	(6.15)	(3.26)	(4.85)	(3.03)	(5.75)	(4.44)
Mean at baseline – control group	2.14	0.56	3.18	2.20	28.66	8.04	6.80	2.55	42.05	13.27
	(1.87)	(0.85)	(2.15)	(2.36)	(45.27)	(27.22)	(25.21)	(15.80)	(49.42)	(33.97)

Number of obs. (N)	759	759	759	759	759	759	759	759	759	759
p-value	0.29	0.37	0.57	0.95	0.19	0.84	0.27	0.30	0.31	0.29
*Production*	Total production-men (kg)	Total production-women (kg)	Grains and tubers-men (kg)	Grains and tubers-women (kg)	Legumes, nuts and pulses-men (kg)	Legumes, nuts and pulses-women (kg)	Other fruits and vegetables-men (kg)	Other fruits and vegetables-women (kg)	Vitamin A-rich crops-men (kg)	Vitamin A-rich crops-women (kg)
Treatment	115.46	46.14	−27.61	−17.38	114.44	15.61	−0.62	3.77	−0.07	0.69
	(260.08)	(85.53)	(195.60)	(49.83)	(77.72)	(36.44)	(42.44)	(4.07)	(28.88)	(0.77)
Mean at baseline – control group	1,790.9 (2841.5)	269.69 (510.70)	1014.9 (2016.7)	149.07 (410.60)	56.87 (204.23)	47.57 (168.69)	1.88 (31.80)	3.21 (21.83)	7.39 (96.34)	0.06 (0.58)

Number of obs. (N)	759	759	759	759	759	759	759	759	759	759
p-value	0.66	0.59	0.89	0.73	0.15	0.67	0.99	0.36	1.00	0.37

Notes: Comparison is to a control group that did not receive any program services. All estimates control for clustering and attrition. Values are coefficients (SE) or mean (SD). * p < 0.10; ** p < 0.05; *** p < 0.01.

In terms of health and nutrition-related outcomes, we found a significant impact of the EHFP program on improving mothers’ health and nutrition-related knowledge in both treatment groups compared to the control group during the program implementation period (Table A5 in Supplementary material). Treatment effects for the post-implementation period suggests that mothers’ knowledge deteriorated after the program implementation, though overall knowledge gains were significant and substantial for the three-year period (Table 2-Panel A and B). The only statistically significant declines were with respect to knowledge of vitamin A- rich foods like dark, green leafy vegetables in the HC group and eggs in both the HC and OWL groups. Overall program effects including one-year post-implementation on mother’s health and nutrition-related knowledge remained significant and ranged from 13 to 24 percentage point change in the OWL group and 12–21 percentage point change in the HC group (Table 2-Panel B).

**Table 2:** Feeding Practices and Health Care Knowledge Effects of Behavior Change Communication Interventions.

Panel A: Post-program impact on beneficiary households, 2012-2013									
*Feeding Practices*	Children should be breastfed less than one hour after birth	Give colostrum to children	Children <6 months of age should not drink any liquids other than breast milk	Begin giving liquids other than breast milk at 6 months of age	Begin giving semi-solid foods at 6 months of age	Vitamin A-rich foods - Orange and yellow fruits and vegetables	Vitamin A-rich foods - Dark green leafy vegetables	Vitamin A-rich foods - Eggs	Vitamin A-rich foods - Liver
OWL group	−0.11	−0.06***	−0.15	−0.05	−0.02	−0.12	−0.10	−0.23***	0.06
	(0.08)	(0.02)	(0.11)	(0.06)	(0.07)	(0.08)	(0.07)	(0.09)	(0.06)
HC group	−0.16**	−0.03	−0.09	−0.10	−0.04	−0.08	−0.15**	−0.13*	−0.01
	(0.08)	(0.03)	(0.10)	(0.07)	(0.06)	(0.09)	(0.07)	(0.07)	(0.05)
Mean at endline - OWL group	0.85	0.98	0.57	0.76	0.80	0.57	0.65	0.64	0.2
	(0.36)	(0.14)	(0.50)	(0.43)	(0.40)	(0.50)	(0.48)	(0.48)	(0.40)
Mean at endline - HC group	0.89	0.96	0.54	0.79	0.76	0.55	0.63	0.56	0.18
	(0.32)	(0.18)	(0.50)	(0.41)	(0.42)	(0.50)	(0.48)	(0.50)	(0.38)
Mean at endline – control group	0.72	0.88	0.29	0.57	0.63	0.27	0.28	0.33	0.08
	(0.45)	(0.33)	(0.46)	(0.50)	(0.48)	(0.45)	(0.45)	(0.47)	(0.27)
Number of observations (N)	1,013	852	841	851	854	842	842	842	842
p-value	0.15	0.01	0.38	0.34	0.79	0.29	0.07	0.03	0.48
*Health Care Practices*	Washing hands - Before eating	Washing hands - Before feeding a child	Washing hands - After using the toilet	Washing hands - After cleaning a child who has defecated	Treating diarrhea - Give oral rehydration salts	Treating diarrhea - Give traditional medicine	Treating diarrhea - Take to medical center		
OWL group	0.04	0.07	0.00	0.00	0.00	0.06**	−0.06		
	(0.10)	(0.10)	(0.08)	(0.05)	(0.06)	(0.03)	(0.06)		
HC group	−0.11	−0.06	0.00	−0.02	−0.03	0.02	−0.02		
	(0.09)	(0.09)	(0.07)	(0.05)	(0.06)	(0.02)	(0.06)		
Mean at endline - OWL group	0.63	0.3	0.12	0.03	0.05	0.03	0.92		
	(0.49)	(0.46)	(0.32)	(0.18)	(0.22)	(0.16)	(0.28)		
Mean at endline - HC group	0.71	0.44	0.12	0.05	0.07	0.03	0.92		
	(0.46)	(0.50)	(0.32)	(0.21)	(0.25)	(0.17)	(0.28)		
Mean at endline – control group	0.65	0.34	0.09	0.06	0.03	0.06	0.91		
	(0.48)	(0.47)	(0.28)	(0.24)	(0.18)	(0.23)	(0.29)		
Number of observations (N)	830	830	830	830	830	830	830		
p-value	0.35	0.41	1.00	0.81	0.88	0.08	0.61		

Panel B: Overall impact on beneficiary households, 2010-2013									
*Feeding Practices*	Children should be breastfed less than one hour after birth	Give colostrum to children	Children <6 months of age should not drink any liquids other than breast milk	Begin giving liquids other than breast milk at 6 months of age	Begin giving semi-solid foods at 6 months of age	Vitamin A-rich foods - Orange and yellow fruits and vegetables	Vitamin A-rich foods - Dark green leafy vegetables	Vitamin A-rich foods - Eggs	Vitamin A-rich foods - Liver
OWL group	0.04	0.20***	0.13*	0.24***	0.17**	0.13*	0.23***	0.06	0.19***
	(0.06)	(0.05)	(0.07)	(0.09)	(0.07)	(0.07)	(0.08)	(0.07)	(0.06)
HC group	0.02	0.12***	0.14**	0.21**	0.13**	0.15**	0.13	0.09	0.08*
	(0.07)	(0.04)	(0.06)	(0.08)	(0.06)	(0.07)	(0.08)	(0.06)	(0.05)
Mean at baseline - OWL group	0.46	0.65	0.20	0.32	0.38	0.23	0.21	0.18	0.02
	(0.50)	(0.48)	(0.40)	(0.47)	(0.49)	(0.42)	(0.41)	(0.38)	(0.14)
Mean at baseline - HC group	0.47	0.74	0.22	0.34	0.37	0.22	0.24	0.17	0.03
	(0.50)	(0.44)	(0.41)	(0.47)	(0.48)	(0.42)	(0.43)	(0.38)	(0.16)
Mean at baseline – control group	0.48	0.80	0.20	0.42	0.41	0.19	0.18	0.16	0.03
	(0.50)	(0.40)	(0.40)	(0.49)	(0.49)	(0.39)	(0.38)	(0.37)	(0.16)
Number of observations (N)	1,013	852	825	851	854	842	842	842	842
p-value	0.84	0.00	0.07	0.01	0.02	0.07	0.02	0.38	0.01
*Health Care Practices*	Washing hands - Before eating	Washing hands - Before feeding a child	Washing hands - After using the toilet	Washing hands - After cleaning a child who has defecated	Treating diarrhea - Give oral rehydration salts	Treating diarrhea - Give traditional medicine	Treating diarrhea - Take to medical center		
OWL group	0.14**	0.17**	0.04	−0.11	−0.03	−0.09**	0.04		
	(0.06)	(0.07)	(0.09)	(0.07)	(0.09)	(0.04)	(0.08)		
HC group	−0.01	0.14*	−0.03	−0.06	−0.07	−0.05	0.13**		
	(0.07)	(0.08)	(0.09)	(0.07)	(0.07)	(0.04)	(0.07)		
Mean at baseline - OWL group	0.48	0.30	0.14	0.23	0.10	0.25	0.83		
	(0.50)	(0.46)	(0.35)	(0.42)	(0.30)	(0.43)	(0.38)		
Mean at baseline - HC group	0.56	0.34	0.22	0.17	0.13	0.17	0.77		
	(0.50)	(0.47)	(0.41)	(0.38)	(0.34)	(0.38)	(0.42)		
Mean at baseline – control group	0.61	0.45	0.16	0.14	0.05	0.13	0.91		
	(0.49)	(0.50)	(0.37)	(0.35)	(0.21)	(0.33)	(0.28)		
Number of observations (N)	830	830	830	830	830	830	830		
p-value	0.08	0.06	0.58	0.30	0.59	0.12	0.14		

Panel C: Post-program impact on new cohort households, 2012-2013									
*Feeding Practices*	Children should be breastfed less than one hour after birth	Give colostrum to children	Children <6 months of age should not drink any liquids other than breast milk	Begin giving liquids other than breast milk at 6 months of age	Begin giving semi-solid foods at 6 months of age	Vitamin A-rich foods - Orange and yellow fruits and vegetables	Vitamin A-rich foods - Dark green leafy vegetables	Vitamin A-rich foods - Eggs	Vitamin A-rich foods - Liver
OWL group	−0.08	−0.06	0.06	0.11	−0.11	0.00	0.06	0.04	−0.03
	(0.08)	(0.06)	(0.09)	(0.07)	(0.08)	(0.06)	(0.06)	(0.08)	(0.07)
HC group	−0.03	−0.05	−0.01	0.00	−0.09	0.04	0.04	0.05	−0.02
	(0.07)	(0.05)	(0.07)	(0.06)	(0.08)	(0.06)	(0.07)	(0.10)	(0.08)
Mean at endline - OWL group	0.81	0.89	0.48	0.66	0.71	0.42	0.47	0.39	0.16
	(0.39)	(0.31)	(0.50)	(0.48)	(0.46)	(0.50)	(0.50)	(0.49)	(0.37)
Mean at endline - HC group	0.79	0.91	0.43	0.72	0.64	0.35	0.40	0.44	0.13
	(0.41)	(0.28)	(0.50)	(0.45)	(0.48)	(0.48)	(0.49)	(0.50)	(0.34)
Mean at endline – control group	0.75	0.84	0.28	0.59	0.59	0.27	0.27	0.33	0.05
	(0.44)	(0.36)	(0.45)	(0.49)	(0.49)	(0.44)	(0.44)	(0.47)	(0.23)
Number of observations (N)	852	764	762	762	762	764	764	764	764
p-value	0.58	0.53	0.69	0.24	0.30	0.79	0.55	0.85	0.92
*Health Care Practices*	Washing hands - Before eating	Washing hands - Before feeding a child	Washing hands - After using the toilet	Washing hands - After cleaning a child who has defecated	Treating diarrhea - Give oral rehydration salts	Treating diarrhea - Give traditional medicine	Treating diarrhea - Take to medical center		
OWL group	−0.04	0.04	−0.03	−0.06	0.02	−0.10***	−0.04		
	(0.11)	(0.10)	(0.06)	(0.05)	(0.04)	(0.03)	(0.05)		
HC group	0.02	0.00	0.02	−0.01	0.10**	−0.11***	−0.02		
	(0.12)	(0.11)	(0.06)	(0.05)	(0.04)	(0.04)	(0.05)		
Mean at endline - OWL group	0.62	0.29	0.10	0.09	0.03	0.04	0.92		
	(0.49)	(0.46)	(0.30)	(0.29)	(0.17)	(0.19)	(0.28)		
Mean at endline - HC group	0.59	0.41	0.05	0.07	0.02	0.07	0.89		
	(0.49)	(0.49)	(0.22)	(0.26)	(0.13)	(0.26)	(0.31)		
Mean at endline – control group	0.61	0.30	0.09	0.05	0.03	0.04	0.88		
	(0.49)	(0.46)	(0.28)	(0.22)	(0.17)	(0.19)	(0.32)		
Number of observations (N)	540	540	540	540	542	542	542		
p-value	0.88	0.89	0.68	0.57	0.06	0.01	0.77		

Notes: Treatment groups are older women leaders (OWL) and health committee members (HC). Comparison is to a control group that did not receive any program services. All estimates control for clustering and attrition. Values are coefficients (SE) or mean (SD). * p < 0.10; ** p < 0.05; *** p < 0.01.

Following on the improvements in mothers’ knowledge, our analysis of birth cohorts at endline indicated that mothers living in OWL villages were more likely to have exclusively breastfed children less than 6 months old as compared to mothers living in control villages (Table 3- Panel A). However, these estimates are suggestive as they do not track the same cohort of birth mothers over time, so we cannot rule out that the composition of the endline group may have also influenced some of the measured differences in IYCF practices. A significant between cohort spillover effect was also measured among non-treated mothers in the new cohort OWL villages. We estimate an 23 pp increase in exclusive breastfeeding children less than 6 months of age and a 2.4 pp reduction in feeding a child using a bottle as compared to mothers in control villages (Table 3- Panel B).

**Table 3:** Panel A: Infant and young child feeding practices among caregivers of children 3–12 months of age at baseline or endline in treated households.

	Breastfeeding					Complementary feeding
	Ever breastfed	Began breastfeeding <1 hour after birth	Exclusively breastfed child <6 months of age	Predominantly breastfed child <6 months of age	Give child a bottle	Give complementary food to children 6-8 months of age
Time	0.18	–12.26**	17.33*	1.43//	–0.78*	26.39***
	(0.14)	(6.05)	(9.21)	(5.32)	(0.43)	(8.16)
OWL group	–0.83	12.35	34.34*	0.38	–0.31	–5.04
	(0.87)	(9.06)	(15.82)	(6.53)	(0.65)	(11.79)
HC group	–0.18	11.98	2.36	–7.66	–0.77	6.10
	(0.14)	(9.77)	(12.41)	(8.50)	(0.88)	(9.57)
Mother’s age (in years)	–0.01	0.01	0.13	0.03	0.03	0.32
	(0.02)	(0.14)	(0.23)	(0.17)	(0.03)	(0.25)
Number of observations (N)	1,874	1,829	736	736	1,883	569
p-value	0.37	0.31	0.08	0.56	0.67	0.51

Panel B: Infant and young child feeding practices among caregivers of children 3-12 months of age at baseline in beneficiary households or 3–12 months at endline in non-treated households in treatment villages							
	Breastfeeding						Complementary feeding
	Ever breastfed	Began breastfeeding <1 hour after birth	Exclusively breastfed child <6 months of age	Predominantly breastfed child <6 months of age	Give child a bottle		Give complementary food to children 6-8 months of age
OWL group	0.11	3.55	22.98**	–0.49	–2.35*		1.57
	(0.41)	(6.83)	(10.78)	(4.12)	(1.30)		(10.51)
HC group	–0.14	1.67	15.53	–4.67	–2.32		–8.37
	(0.33)	(8.35)	(10.24)	(5.40)	(1.69)		(10.34)
Mother’s age (in years)	–0.01	0.07	0.38**	0.06	0.02		0.42**
	(0.01)	(0.12)	(0.18)	(0.16)	(0.04)		(0.21)

Time	0.13	–10.43***	16.47***	2.07	2.12*		17.79**
	(0.33)	(3.98)	(6.08)	(2.97)	(1.12)		(7.63)
Number of observations (N)	2,403	2,358	897	897	2,410		728
p-value	0.57	0.87	0.08	0.67	0.19		0.57

Notes: Treatment groups are older women leaders (OWL) and health committee members (HC). Comparison is to a control group that did not receive any program services. All estimates are controlled for mother’s age, clustering, and attrition. All values are coefficient (SE). * p < 0.10; ** p < 0.05; *** p < 0.01.

In any integrated agriculture and nutrition intervention targeted to children, the primary outcomes of interest are children’s nutritional status indicators. The primary findings during the program implementation period indicated improved hemoglobin concentration, and reductions in the prevalence of anemia and, wasting among children living in HC villages relative to those living in control villages (Table A5 in Supplementary material). In the post-implementation period, no further increases in hemoglobin concentrations were measured or reductions in the prevalence of anemia or severe anemia (Table 4-Panel A).

**Table 4:** Nutrition and Anemia Outcomes from Agricultural and Behavior Change Communication Interventions.

Panel A: Post-program impact on treated households with children aged 3-12 months at baseline, 2012-2013										
	Hemo-globin (g/dL)	Anemic (Hb<11 g/dL)	Severely anemic (Hb<7 g/dL)	Hemo-globin (g/dL) (3-5.9 mo)	Anemic (Hb<11 g/dL) (3-5.9 mo)	Severely anemic (Hb<7 g/DL) (3-5.9 mo)	Weight-for-age Z-score (WAZ)	Weight-for-height Z-score (WHZ)	Under-weight (WAZ<-2)	Wasted (WHZ<-2)
OWL group	0.16	−0.03	−0.02	0.22	0.06	−0.01	0.03	0.06	0.01	0.02
	(0.24)	(0.07)	(0.02)	(0.33)	(0.08)	(0.03)	(0.11)	(0.14)	(0.04)	(0.03)
HC group	−0.15	0.01	0.00	−0.35	0.08	−0.01	0.04	0.07	0.01	−0.02
	(0.17)	(0.05)	(0.01)	(0.32)	(0.09)	(0.02)	(0.08)	(0.09)	(0.04)	(0.03)
Mean at endline - OWL group	9.64	0.81	0.03	9.49	0.80	0.04	−1.41	−0.69	0.25	0.09
	(1.55)	(0.40)	(0.18)	(1.66)	(0.40)	(0.19)	(1.03)	(0.98)	(0.43)	(0.28)
Mean at endline - HC group	9.93	0.75	0.03	9.92	0.75	0.03	−1.65	−0.76	0.32	0.08
	(1.43)	(0.43)	(0.16)	(1.53)	(0.43)	(0.18)	(1.00)	(1.01)	(0.47)	(0.28)
Mean at endline – control group	9.72	0.80	0.03	9.59	0.85	0.02	−1.47	−0.60	0.29	0.09
	(1.47)	(0.40)	(0.16)	(1.47)	(0.36)	(0.16)	(1.12)	(1.27)	(0.45)	(0.29)
Number of observations (N)	940	940	940	385	385	385	707	690	707	690
p-value	0.43	0.81	0.74	0.41	0.55	0.77	0.90	0.71	0.93	0.39

Panel B: Overall impact on treated households with children aged 3-12 months at baseline, 2010-2013										
	Hemo-globin (g/dL)	Anemic (Hb<11 g/dL)	Severely anemic (Hb<7 g/dL)	Hemo-globin (g/dL) (3-5.9 mo)	Anemic (Hb<11 g/dL) (3-5.9 mo)	Severely anemic (Hb<7 g/DL) (3-5.9 mo)	Weight-for-age Z-score (WAZ)	Weight-for-height Z-score (WHZ)	Under- weight (WAZ<-2)	Wasted (WHZ<-2)
OWL group	0.31	0.00	−0.07	0.16	0.05	−0.04	−0.22	0.00	0.09	0.04
	(0.38)	(0.07)	(0.06)	(0.35)	(0.09)	(0.04)	(0.16)	(0.16)	(0.06)	(0.05)
HC group	0.45	−0.02	−0.05*	0.60*	−0.06	−0.09*	0.07	0.26	0.05	−0.05
	(0.27)	(0.06)	(0.03)	(0.31)	(0.07)	(0.05)	(0.15)	(0.16)	(0.06)	(0.05)
Mean at baseline - OWL group	8.90	0.86	0.15	9.34	0.84	0.10	−1.33	−0.94	0.29	0.24
	(1.88)	(0.34)	(0.36)	(1.74)	(0.37)	(0.30)	(1.55)	(1.77)	(0.46)	(0.43)
Mean at baseline - HC group	8.81	0.87	0.14	8.84	0.89	0.14	−1.66	−1.18	0.38	0.30
	(1.74)	(0.34)	(0.35)	(1.76)	(0.32)	(0.35)	(1.57)	(1.80)	(0.49)	(0.46)
Mean at baseline – control group	9.14	0.89	0.09	9.38	0.86	0.07	−1.56	−0.94	0.39	0.24
	(1.69)	(0.32)	(0.29)	(1.71)	(0.35)	(0.25)	(1.71)	(1.81)	(0.49)	(0.43)
Number of observations (N)	940	940	940	385	385	385	778	754	778	754
p-value	0.26	0.95	0.15	0.15	0.43	0.24	0.28	0.13	0.31	0.29

Panel C: Post-program impact on treated households with children aged 3-12 months at endline, 2012-2013										
	Hemo-globin (g/dL)	Anemic (Hb<11 g/dL)	Severely anemic (Hb<7 g/dL)	Hemo-globin (g/dL) (3-5.9 mo)	Anemic (Hb<11 g/dL) (3-5.9 mo)	Severely anemic (Hb<7 g/DL) (3-5.9 mo)	Weight-for-age Z-score (WAZ)	Weight-for-height Z-score (WHZ)	Under-weight (WAZ<-2)	Wasted (WHZ<-2)
OWL group	−0.03	0.01	0.02	−0.15	0.17	0.00	−0.30	−0.25	0.06	−0.20***
	(0.44)	(0.09)	(0.05)	(0.50)	(0.11)	(0.08)	(0.33)	(0.42)	(0.13)	(0.07)
HC group	−0.15	0.11**	0.02	−0.66	0.18*	0.01	−0.20	0.19	−0.07	−0.20***
	(0.27)	(0.05)	(0.04)	(0.49)	(0.11)	(0.07)	(0.32)	(0.28)	(0.09)	(0.07)
Mean at endline - OWL group	9.48	0.91	0.01	9.76	0.82	0.00	−1.15	−0.50	0.23	0.13
	(1.47)	(0.29)	(0.11)	(1.67)	(0.39)	(0.00)	(1.55)	(1.61)	(0.43)	(0.34)
Mean at endline - HC group	9.36	0.86	0.02	9.97	0.75	0.02	−1.26	−0.90	0.31	0.21
	(1.33)	(0.34)	(0.13)	(1.30)	(0.44)	(0.13)	(1.64)	(1.66)	(0.47)	(0.41)
Mean at endline – control group	9.12	0.96	0.05	9.36	0.95	0.05	−1.17	−0.69	0.24	0.16
	(1.29)	(0.21)	(0.21)	(1.29)	(0.22)	(0.22)	(1.41)	(1.56)	(0.43)	(0.37)
Number of observations (N)	231	231	231	97	97	97	188	184	188	184
p-value	0.84	0.11	0.89	0.37	0.09	0.97	0.66	0.59	0.56	0.01

Panel D: Post-program impact on non-treated new cohort households with children aged 3-12 months at endline. 2012-2013										
	Hemo-globin (g/dL)	Anemic (Hb<7 g/dL)	Severely anemic (Hb<11 g/dL)	Hemo-globin (g/dL) (3-5.9 mo)	Anemic (Hb<11 g/dL) (3-5.9 mo)	Severely anemic (Hb<7 g/DL) (3-5.9 mo)	Weight-for-age Z-score (WAZ)	Weight-for-height Z-score (WHZ)	Under-weight (WAZ<-2)	Wasted (WHZ<-2)
OWL group	−0.26	0.04	−0.01	−0.19	0.05	−0.06	−0.06	−0.08	0.08	−0.04
	(0.23)	(0.04)	(0.03)	(0.30)	(0.07)	(0.04)	(0.12)	(0.18)	(0.06)	(0.06)
HC group	0.05	−0.01	−0.03	−0.17	0.05	−0.02	0.10	0.19	0.03	0.01
	(0.22)	(0.05)	(0.04)	(0.28)	(0.06)	(0.05)	(0.15)	(0.13)	(0.04)	(0.04)
Mean at endline – OWL group	9.33	0.87	0.04	9.74	0.80	0.01	−1.41	−1.07	0.30	0.23
	(1.45)	(0.33)	(0.20)	(1.47)	(0.40)	(0.11)	(1.25)	(1.25)	(0.46)	(0.42)
Mean at endline – HC group	9.24	0.91	0.07	9.61	0.84	0.05	−1.32	−0.99	0.28	0.19
	(1.31)	(0.29)	(0.26)	(1.35)	(0.37)	(0.21)	(1.51)	(1.27)	(0.45)	(0.39)
Mean at endline – control group	9.10	0.92	0.06	9.46	0.89	0.03	−1.37	−0.93	0.31	0.18
	(1.32)	(0.26)	(0.23)	(1.30)	(0.32)	(0.18)	(1.37)	(1.41)	(0.47)	(0.38)
Number of observations (N)	630	630	630	238	238	238	553	538	553	538
p-value	0.45	0.55	0.74	0.75	0.67	0.26	0.58	0.21	0.39	0.67

Panel E: Within village cohort program impact on treated households with children 3–12 months at baseline or endline, 2010-2012							
	Hemoglobin (g/dL)	Anemic (Hb<11 g/dL)	Severely anemic (Hb<7 g/dL)	Weight-for-age Z-score (WAZ)	Weight-for-height Z-score (WHZ)	Underweight (WAZ<-2)	Wasted (WHZ<-2)
OWL group (1 = 2012 OWL cohort)	0.27	−3.05	−4.71	−0.13	−0.37	14.75**	2.84
	(0.26)	(5.39)	(3.72)	(0.24)	(0.36)	(6.79)	(7.79)
HC group (1 = 2012 HC cohort)	0.38	−6.12	−7.12***	0.11	0.01	2.85	−5.43
	(0.28)	(5.83)	(2.58)	(0.25)	(0.48)	(7.16)	(6.29)
Child’s age (in months)	−0.10***	0.74***	1.24***	−0.15***	−0.05	3.36***	0.89**
	(0.01)	(0.21)	(0.25)	(0.02)	(0.05)	(0.47)	(0.39)
Child sex (1=boy)	−0.12*	0.25	1.23	−0.41***	−0.32	12.52***	4.09**
	(0.07)	(1.30)	(1.65)	(0.08)	(0.20)	(2.09)	(1.82)
Number of observations (N)	2,223	2,223	2,223	2,224	2,224	2,224	2,224
p-value	0.29	0.58	0.02	0.67	0.57	0.10	0.58

Notes: Treatment groups are older women leaders (OWL) and health committee members (HC). Comparison is to a control group that did not receive any program services. A year fixed effect is included in the specification. Standard errors are clustered at the village level. All regressions use attrition weights. * p < 0.10; ** p < 0.05; *** p < 0.01.

In Table 4-Panel B, most of these nutrition impacts were sustained one year after the end of program implementation. Children 3–12 months of age at baseline living in HC villages were 5 percentage points less likely to be severely anemic as compared to those living in control villages. Among children 3–5.9 months of age at baseline living in HC villages, hemoglobin concentration was higher, and the prevalence of severe anemia was 9 percentage points lower compared to children 3–5.9 months of age living in control villages.

To measure spillovers within households, the effect of the intervention on younger siblings within treated households is estimated in Table 4-Panel C. Among the younger siblings of the target children (children 3–12 months of age in beneficiary households in 2012), there was a protective effect of the EHFP program on the normal increase in the prevalence of wasting that is seen in this population over the first two years of life. Whereas the prevalence of wasting increased from 2012 to 2013 among children that were 3–12 months of age in 2012 in control villages, in OWL villages there was no change and in HC villages there was a decline, resulting in a 20 percentage point difference in each of the treatment groups and the control group. These differences were statistically significant and indicate a potential role of the EHFP program in protecting children’s nutritional status during this vulnerable age. We observe a rise in anemia in siblings of HC treated children (11 pp increase) and among siblings of HC treated children 3–5.9 months (18 pp increase), though the full sample treatment effect is not statistically different than zero (p-value = 0.11).

No other spillover effects were measured for the new cohort of children with respect to their nutritional status estimating the spillover using a between village specification (Table 4-Panel D). Estimating within village cohort spillover effects, non-treated children living in HC villages were 7 percentage points less likely to be severely anemic compared to treated children assessed at baseline (Table 4-Panel E).

### 4.3 Robustness of main results to multiple hypothesis testing corrections

As we estimate different treatment effects over varying time periods and multiple spillover effects, Table 5 presents a summary of the main statistically significant results by time period, noting the effect size and pvalue of the null hypothesis that the coefficient estimate is statistically different than zero for statistically significant treatment effects. Table 5 also presents the corrected q-values for multiple hypothesis testing following Anderson (2008).

**Table 5:** Summary of Estimated Effects and Multiple Hypothesis Robustness.

	Post-program Impacts (2012-2013)				Overall impact (2010-2013)				Spillover effects			
	Effect size	Treatment Group	p-value	q-value	Effect size	Treatment Group	p-value	q-value	Effect size	Treatment Group	p-value	q-value
Agriculture												
*Land*												
Number of plots-women					56%	Pooled	0.02	0.041				
*Inputs*												
Fertilizer use-women (%)	−5%	Pooled	0.06	0.091								
Manure use -women (%)	−27%	Pooled	0.00	0.001	21%	Pooled	0	0.001				
Knowledge												
*Breastfeeding*												
Children should be breastfed less than one hour after birth												
Give colostrum to children	−6%	OWL	0.01	0.051	20%	OWL	0	0.001				
					12%	HC						
Children <6 months of age should not drink any liquids other than breast milk					13%	OWL	0.07	0.088				
					14%	HC						
Begin giving liquids other than breast milk at 6 months of age					24%	OWL	0.01	0.026				
					21%	HC						
Begin giving semi-solid foods at 6 months of age					17%	OWL	0.02	0.034				
					13%	HC						
*Nutrient-rich foods*												
Vitamin A-rich foods - Orange and yellow fruits and vegetables					13%	OWL	0.07	0.094				
Vitamin A-rich foods - Orange and yellow fruits and vegetables					15%	HC						
Vitamin A-rich foods - Dark green leafy vegetables	−15%	HC	0.07	0.14	23%	OWL	0.02	0.041				
Vitamin A-rich foods - Eggs	−23%	OWL	0.03	0.121								
Vitamin A-rich foods - Eggs	−13%	HC										
	Post-program Impacts (2012-2013)				Overall impact (2010-2013)				Spillover effects			
	Effect size	Treatment Group	p-value	q-value	Effect size	Treatment Group	p-value	q-value	Effect size	Treatment Group	p-value	q-value
Vitamin A-rich foods - Liver					19%	OWL	0.01	0.041				
					8%	HC						
*Health care practices*												
Washing hands - Before eating					14%	OWL	0.08	0.161				
Washing hands - Before feeding a child					17%	OWL	0.06	0.161				
					14%	HC						
Treating diarrhea - Give rehydration salts									10%	HC	0.06	0.091
Treating diarrhea - Give traditional medicine	6%	OWL	0.08	0.241					−10%	OWL	0.01	0.031
									−11%	HC		
Practices												
*Breastfeeding*												
Exclusively breastfed child <6 months of age									34%	OWL (Within)	0.08	0.401
									23%	OWL (Between)	0.08	0.401
Nutritional Outcomes												
*Anemia*												
Anemic (Hb<11 g/dL) (3-5.9 mo)									18%	HC (Between, Siblings)	0.09	0.27
Severely anemic (Hb<7 g/dL)					−5%	HC	0.15	0.391	−7%	HC (Within, siblings)	0.02	0.061
Hemoglobin (g/dL) (3-5.9 mo)					.6	HC	0.15	0.361				
Severely anemic (Hb<11 g/dL) (3-5.9 mo)					−9%	HC	0.24	0.361				
*Wasting*												
Wasted (WHZ<-2)									−20%	OWL, HC (Between, Siblings)	0.01	0.021

Notes: This table summarizes study results that were statistically significant in the study’s main tables. The effect size and p-value of the null hypothesis that the effect is statistically different than zero are reported. The q-value presented is the estimate of the multiple hypothesis correction for the p-value following Anderson et al. (2017). All spillover effects reported are household spillovers with the exception of the nutritional outcomes reported in Table.

The overall program effects are robust to multiple hypothesis testing (15 out of 21 statistically significant results where p-values rejected the null hypothesis that the treatment effect was equal to zero). The sustained impacts on anemia and hand washing were not robust to multiple hypothesis testing, while the results on women’s agricultural land holdings, inputs, breastfeeding knowledge, and nutrient rich food knowledge were robust to multiple hypothesis testing.

Three out of the seven post-program effects are statistically significant after multiple hypothesis corrections. The results which were not robust to multiple hypothesis testing were the results on mother’s knowledge of vitamin A rich foods (dark leafy vegetables and eggs) and treating diarrhea using traditional medicine. Results on agricultural inputs and breastfeeding knowledge were robust to multiple hypothesis testing among the post-program effects.

Five out of the eight statistically significant spillover effects estimated are also robust to multiple hypothesis correction. The results that were not robust to multiple hypothesis testing included the within and between cohort results on breastfeeding and the anomalous result on increased anemia prevalence among younger siblings. The spillover effects on the treatment of diarrhea, anemia and wasting remained robust to multiple hypothesis testing.

## 5 Conclusion

This paper demonstrates the importance of post-program effects in evaluating the impact of an integrated agriculture and nutrition program where disadoption dynamics, knowledge deterioration or spillovers may affect overall program impact assessment. Despite deterioration of some of the program implementation period effects and limited production and knowledge spillover effects, we found a significant impact of an agriculture and nutrition program on improving children’s hemoglobin concentration during the program period and sustained impacts one-year after the program ended. We also demonstrate a potential protective effect of the program on younger children born into beneficiary households during the program period on preventing wasting during the first two years of life. While no impacts across treatment cohorts were estimated on children’s anthropometric outcomes, within HC villages, younger children were statistically less likely to be severely anemic.

Production or nutrition BCC mechanisms provided some explanation for these sustained and spillover effects. We found small effects from increased agricultural production over the program implementation period and limited spillovers to non-treated women from production interventions in the post-program implementation period. Knowledge related to some optimal health and nutrition practices deteriorated during the post-implementation period, though it remained significantly higher than knowledge among mothers in control villages over the three-year study period. Overall, program spillovers are limited with respect to health and nutrition-related knowledge, except for an important decline in the belief that traditional medicine should be used to treat children’s diarrhea. Finally, we found a statistically significant child feeding spillover effect on exclusively breastfeeding children under 6 months of age, though this result is not robust to multiple hypothesis testing corrections.

Despite significant positive effects during the program implementation period on hemoglobin concentrations, several factors may explain the lack of impact on other nutritional outcomes with respect to the between village spillovers. First, agricultural production impacts one year after program implementation were not detected in our sample. Given these disadoption dynamics, availability of nutrient-rich foods did not significantly change for beneficiary households or non-beneficiary households in treatment villages. Without program support, the agricultural component implemented over the two-year project was not able to sufficiently resolve the production constraints including access to water during the dry season, faced by rural Burkinabe producers. Second, agricultural production requires labor that women with young children may not have been able to sustain after the initial program period. Without program monitoring, the incentives to provide labor may have decreased, resulting in lower production.

With respect to the role of nutrition education in sustaining program impacts or promoting spillovers, the impact estimates clearly show that the modality of providing nutrition education either through health committees or drawing on the experience of older women leaders was critical to sustaining impacts and spillovers within beneficiary households and across cohorts. In Olney et al. (2015), initial program period effects are likely explained by the mixed gender health committee’s effectiveness in communicating nutrition messages, but also potentially by having more technical health knowledge to connect mothers and children to the formal health system. If this is the case, sustained effects and spillovers are potentially explained through the program’s mobilization and demonstration of new roles for the health committee and OWLs in monitoring and providing information to new mothers. These nutritional resource people including both village health committee leaders and older women leaders remained in treatment communities which could explain the presence of and differences in within village spillovers. Further research on why different types of leaders led to differences in spillovers is important to understanding the role of BCC on nutritional outcomes. The potential integrated connectivity of households within treatment villages either to other households or to those implementing the nutrition education components of the program will be an area of future research which we will explore to further understand the mechanisms driving the anemia and wasting spillover effects we measure in this paper.

## Footnotes

1 In previous work assessing the impact of the EHFP, significant effects on reducing the prevalence of child anemia and wasting, and increasing household consumption of nutrient-rich foods were found (Dillon et al., 2012; Olney et al., 2015).

2 The protocol was approved by the Ministry of Health of Burkina Faso, and the institutional review boards of the International Food Policy Research Institute and Michigan State University.

3 Dillon et al. (2019) outline the multiple mechanisms that could affect child nutrition from the theoretical perspective of an agricultural household model with a health production function.

4 Apart from household attrition which we discuss in detail below, three invited households refused to participate and were not included in the study sample.

5 Alternative experimental designs could also measure spillovers in this context. Baird et al. (2014) discuss an innovative design to randomize the intensity of treatment within clusters and provide an example from Malawi.

6 This method of inverse probability weighting (Wooldridge 2002) only accounts for attrition bias if observable characteristics and not other unobservable characteristics, of the household (motivation to seek health care for their children or willingness to seek jobs in other locations) or child (genetic endowments or health history) influence attrition. While this approach cannot rule out that unobservable characteristics may bias estimates, correcting the estimates based on observable characteristics reduces observable characteristic attrition bias. Implementing an inverse probability weighting correction requires the estimation of the following equation, $P\left(Attrition\right)=\beta X_{baseline}+\gamma District+\varepsilon$. This specification is estimated using household characteristics to estimate the determinants of attrition for households and child and household characteristics to estimate the determinants of attrition for children and includes district level indicators. Using the coefficient estimates from the relevant household or child specification, the probability of attrition is then predicted for each household or child based on their observable characteristics. The inverse of this predicted probability is then used to re-weight the regression estimates to correct for attrition bias.

7 We also conducted the balancing tests using standardized means which produces similar results to those reported in Tables A1 and A2. We report the unadjusted means to provide context and comparability of the study area to other studies.
