# Supplementary material for: Experimental evidence on post-program effects and spillovers from an agriculture-nutrition program
Source: Econ Hum Biol. 2020 Jan;36:100820. doi: 10.1016/j.ehb.2019.100820 (PMC6988509; doi:10.1016/j.ehb.2019.100820)
Supplement: Supplementary file 1 [file mmc1.pdf]

Appendix to the paper

"Experimental Evidence on Post-Program Effects and Spillovers from an Agriculture-Nutrition Program"

Table A1: Balancing Test of Initial Cohort of Household and Child Characteristics[1]

Variable	All	Control	OWL	HC	p-value
Households (HH) (N)	1,481	597	443	441	
HH size (residents present at least 6 months)	7.72 ± 3.69	8.02 ± 3.82	7.78 ± 3.64	7.24 ± 3.52	0.33
Number of children (under 6 years of age)	2.63 ± 1.46	2.73 ± 1.53	2.62 ± 1.36	2.52 ± 1.48	0.49
Polygamous HH	39.0%	41.7%	40.4%	33.3%	0.33
Female Headed HH	7.0%	6.5%	6.5%	7.1%	0.96
Men's Asset Value (FCFA)[2]	73,661 ± 58,564	72,689 ± 54,694	79,121 ± 61,290	69,470 ± 60,461	0.34
Women's Asset Value (FCFA)[2]	43,053 ± 54,915	44,294 ± 36,923	45,195 ± 50,254	39,234 ± 75,621	0.51
Dirt floor in primary house	41.7%	35.7%	42.9%	48.5%	0.07
Straw roofing in primary house	59.7%	60.3%	61.6%	57.1%	0.70
HH head had any formal education	10.2%	9.8%	11.3%	9.9%	0.89
Mother had any formal education	6.6%	6.6%	5.9%	7.1%	0.85
Children (N)	1,452	577	443	432	
Child's sex (% male)	50.0%	50.6%	48.9%	50.5%	0.79
Child's age (months)	7.26 ± 2.65	7.40 ± 2.64	7.14 ± 2.60	7.21 ± 2.71	0.26
Hemoglobin (g/dL)	8.90 ± 1.71	9.07 ± 1.65	8.77 ± 1.74	8.82 ± 1.75	0.37
Prevalence of anemia (Hb < 11.0 g/dL)	88.9%	89.5%	89.8%	87.2%	0.73
Prevalence of severe anemia (Hb < 7.0 g/dL)	12.9%	10.4%	14.9%	14.3%	0.26
Weight-for-age Z-score (WAZ)	-1.58 ± 1.63	-1.59 ± 1.65	-1.42 ± 1.61	-1.73 ± 1.60	0.05
Prevalence of underweight (WAZ < -2)	37.9%	39.4%	33.1%	41.0%	0.08
Weight-for-height Z-score (WHZ)	-1.03 ± 1.83	-0.96 ± 1.80	-0.97 ± 1.86	-1.18 ± 1.84	0.26
Prevalence of wasting (WHZ < -2)	27.5%	24.9%	26.7%	31.8%	0.32

[1]All values are mean ± SD or percent, [2]FCFA (Franc Communauté Financière Africaine): West African CFA franc

Table A2: Balancing Test of New Cohort of Household and Child Characteristics[1]

Variable	All	Control	OWL	HC	p-value
Households (HH) (N)	825	370	168	287	
HH size (residents present at least 6 months)	7.29±3.93	7.61±4.26	7.23±3.65	6.92±3.62	0.27
Number of children (under 6 years of age)	2.30±1.22	2.37±1.29	2.27±1.24	2.23±1.12	0.52
Polygamous HH	28.85%	32.43%	27.98%	24.74%	0.30
Female Headed HH	13.70%	11.62%	11.90%	17.42%	0.11
Men's Asset Value (FCFA)[2]	58,029±46,869	60,001±51,114	63,236±42,707	52,440±42,905	0.13
Women's Asset Value (FCFA)[2]	48,161±49,282	52,768±53,427	44,389±36,718	44,429±49,776	0.22
Dirt floor in primary house	48.79%	49.59%	47.02%	48.78%	0.93
Straw roof in primary house	50.24%	47.43%	51.79%	52.96%	0.73
HH head had any formal education	11.65%	10.30%	14.88%	11.50%	0.46
Mother had any formal education	9.00%	8.82%	9.04%	9.22%	0.99
Children (N)	1,121	525	223	373	
Child's sex (% male)	49.69%	51.05%	50.67%	47.18%	0.41
Child's age (months)	13.80±12.19	14.13±12.39	13.37±11.93	13.61±12.08	0.69
Hemoglobin (g/dL)	9.32±1.41	9.22±1.39	9.29±1.45	9.48±1.40	0.28
Prevalence of anemia (Hb < 11.0 g/dL)	88.00%	89.51%	88.08%	85.62%	0.34
Prevalence of severe anemia (Hb < 7.0 g/dL)	5.44%	5.97%	4.15%	5.43%	0.56
Weight-for-age Z-score (WAZ)	-1.39±1.33	-1.44±1.28	-1.36±1.19	-1.35±1.47	0.69
Prevalence of underweight (WAZ < -2)	29.68%	31.43%	26.25%	29.18%	0.44
Weight-for-height Z-score (WHZ)	-0.93±1.26	-0.89±1.29	-0.98±1.19	-0.96±1.19	0.64
Prevalence of wasting (WHZ < -2)	17.54%	16.62%	20.00%	17.36%	0.73

[1]All values are mean ± SD or percent, [2]FCFA (Franc Communauté Financière Africaine): West African CFA franc

Table A3: Balancing Test of Initial Cohort vs. New Cohort of Household and Child Characteristics

Variable	Old Cohort	New Cohort	p-value
Households (HH) (N)	1,481	825	
HH size (residents present at least 6 months)	7.72 ± 3.69	7.29 ± 3.93	0.16
Number of children (under 6 years of age)	2.63 ± 1.46	2.30 ± 1.22	0
Polygamous HH	38.83%	28.85%	0
Female Headed HH	6.69%	13.70%	0
Men’s Asset Value (FCFA)2	73,661 ± 58,564	58,030 ± 46,869	0
Women’s Asset Value (FCFA)2	43,053 ± 54,915	48,161 ± 49,283	0.11
Dirt floor in primary house	41.66%	48.79%	0.04
Roofing material is straw mat in primary house	59.76%	50.24%	0
HH head had any formal education	10.24%	11.65%	0.28
Mother had any formal education	6.56%	9.00%	0.07
Children (N)	1,452	683	
Child's sex (% male)	50.07%	48.61%	0.54
Child's age (months)	7.26 ± 2.65	7.35 ± 2.57	0.49
Hemoglobin (g/dL)	8.92 ± 1.71	9.22 ± 1.34	0.01
Prevalence of anemia (Hb < 11.0 g/dL)	88.89%	90.62%	0.37
Prevalence of severe anemia (Hb < 7.0 g/dL)	12.91%	5.72%	0
Height-for-age Z-score (HAZ)	-1.23 ± 1.60	-0.98 ± 1.32	0
Prevalence of stunting (HAZ < -2)	30.73%	19.66%	0
Weight-for-age Z-score (WAZ)	-1.58 ± 1.63	-1.29 ± 1.31	0
Prevalence of underweight (WAZ < -2)	37.90%	27.90%	0
Weight-for-height Z-score (WHZ)	-1.03 ± 1.83	-0.96 ± 1.27	0.38
Wasting (WHZ < -2)	27.46%	18.22%	0

1All values are mean ± SD or percent, 2FCFA (Franc Communauté Financière Africaine): West African CFA franc

Table A4 - Program impact on beneficiary households, 2010-2012

Inputs	Hectares cultivated-men	Hectares cultivated-women	Number of plots - men	Number of plots -women	Fertilizer use-men (%)	Fertilizer use-women (%)	Pesticide/ insecticide/ herbicide use-men (%)	Pesticide/ insecticide/ herbicide use-women (%)	Manure use-men (%)	Manure use-women (%)
Treatment	0.33	-0.45	0.34*	2.29***	-0.39	9.25***	4.69	3.06*	9.65*	46.61***
	(0.24)	(0.39)	(0.20)	(0.43)	(4.16)	(2.84)	(4.14)	(1.68)	(5.66)	(4.74)
Mean at baseline – control group	3.01<br>(2.73)	0.98<br>(4.72)	3.50<br>(1.99)	2.36<br>(2.48)	23.83<br>(42.63)	4.18<br>(20.03)	8.29<br>(27.60)	1.27<br>(11.21)	53.19<br>(49.93)	9.16<br>(28.87)
Number of obs. (N)	1,463	1,463	1,463	1,463	1,463	1,463	1,463	1,463	1,463	1,463
p-value	0.16	0.26	0.10	0.00	0.93	0.00	0.26	0.07	0.09	0.00
Production	Total production -men (kg)	Total production -women (kg)	Grains and tubers-men (kg)	Grains and tubers-women (kg)	Legumes, nuts and pulses-men (kg)	Legumes, nuts and pulses-women (kg)	Other fruits and vegetables-men (kg)	Other fruits and vegetables-women (kg)	Vitamin A-rich crops-men (kg)	Vitamin A-rich crops-women (kg)
Treatment	-113.23	82.83	54.97	57.30**	-2.73	-18.68	-19.15*	-2.56	11.35	2.84*
	(200.37)	(64.65)	(113.70)	(22.72)	(27.10)	(20.35)	(11.44)	(7.00)	(13.05)	(1.66)
Mean at baseline – control group	604.00<br>(647.00)	108.00<br>(248.00)	391.00<br>(573.00)	38.00<br>(157.00)	23.07<br>(69.00)	21.91<br>(99.00)	4.70<br>(57.00)	1.74<br>(16.00)	8.51<br>(139.00)	0.18<br>(4.40)
Number of obs. (N)	1,463	1,463	1,463	1,463	1,463	1,463	1,463	1,463	1,463	1,463
p-value	0.57	0.21	0.63	0.01	0.92	0.36	0.10	0.72	0.39	0.09

Notes: Comparison is to a control group that did not receive any program services. All estimates control for clustering and attrition. Values are coefficients (SE) or mean (SD). * $p < 0.10$; ** $p < 0.05$; *** $p < 0.01$.

Table A5 - Program impact on beneficiary households, 2010-2012

Feeding Practices	Children should be breastfed less than one hour after birth	Give colostrum to children	Children <6 months of age should not drink any liquids other than breast milk	Begin giving liquids other than breast milk at 6 months of age	Begin giving semi-solid foods at 6 months of age	Vitamin A-rich foods - Orange and yellow fruits and vegetables	Vitamin A-rich foods - Dark green leafy vegetables	Vitamin A-rich foods - Eggs	Vitamin A-rich foods - Liver
OWL group	0.15	0.26***	0.28***	0.29***	0.19***	0.25***	0.34***	0.30***	0.13***
	(0.11)	(0.06)	(0.11)	(0.07)	(0.06)	(0.07)	(0.05)	(0.05)	(0.04)
HC group	0.18*	0.15***	0.23**	0.30***	0.17***	0.24***	0.28***	0.22***	0.10***
	(0.10)	(0.04)	(0.10)	(0.06)	(0.07)	(0.05)	(0.06)	(0.05)	(0.03)
Mean at baseline - OWL group	0.46	0.65	0.20	0.32	0.38	0.23	0.21	0.18	0.02
	(0.50)	(0.48)	(0.40)	(0.47)	(0.49)	(0.42)	(0.41)	(0.38)	(0.14)
Mean at baseline - HC group	0.47	0.74	0.22	0.34	0.37	0.22	0.24	0.17	0.03
	(0.50)	(0.44)	(0.41)	(0.47)	(0.48)	(0.42)	(0.43)	(0.38)	(0.16)
Mean at baseline – control group	0.48	0.80	0.20	0.42	0.41	0.19	0.18	0.16	0.03
	(0.50)	(0.40)	(0.40)	(0.49)	(0.49)	(0.39)	(0.38)	(0.37)	(0.16)
Number of observations (N)	1,013	852	825	851	854	842	842	842	842
p-value	0.25	0.00	0.03	0.00	0.01	0.00	0.00	0.00	0.00

Health Care Practices	Washing hands - Before eating	Washing hands - Before feeding a child	Washing hands - After using the toilet	Washing hands - After cleaning a child who has defecated	Treating diarrhea - Give oral rehydration salts	Treating diarrhea - Give traditional medicine	Treating diarrhea - Take to medical center
OWL group	0.10	0.11	0.05	-0.12*	-0.03	-0.15***	0.09*
	(0.07)	(0.08)	(0.04)	(0.07)	(0.06)	(0.05)	(0.05)
HC group	0.10	0.21**	-0.03	-0.04	-0.05	-0.07**	0.16***
	(0.07)	(0.09)	(0.04)	(0.05)	(0.04)	(0.04)	(0.05)
Mean at baseline - OWL group	0.48	0.30	0.14	0.23	0.10	0.25	0.83
	(0.50)	(0.46)	(0.35)	(0.42)	(0.30)	(0.43)	(0.38)
Mean at baseline - HC group	0.56	0.34	0.22	0.17	0.13	0.17	0.77
	(0.50)	(0.47)	(0.41)	(0.38)	(0.34)	(0.38)	(0.42)
Mean at baseline – control group	0.61	0.45	0.16	0.14	0.05	0.13	0.91
	(0.49)	(0.50)	(0.37)	(0.35)	(0.21)	(0.33)	(0.28)
Number of observations (N)	830	830	830	830	830	830	830
p-value	0.29	0.06	0.19	0.22	0.50	0.00	0.01

Notes: Treatment groups are older women leaders (OWL) and health committee members (HC). Comparison is to a control group that did not receive any program services. All estimates control for clustering and attrition. Values are coefficients (SE) or mean (SD). * $p < 0.10$; ** $p < 0.05$; *** $p < 0.01$.

Table A6 - Program impact on treated households with children aged 3-12 months at baseline, 2010-2012

	Hemo-globin (g/dL)	Anemic (Hb<11 g/dL)	Severely anemic (Hb<7 g/dL)	Hemo-globin (g/dL) (3-5.9 mo)	Anemic (Hb<11 g/dL) (3-5.9 mo)	Severely anemic (Hb<7 g/DL) (3-5.9 mo)	Weight-for-age Z-score (WAZ)	Weight-for-height Z-score (WHZ)	Under-weight (WAZ<-2)	Wasted (WHZ<-2)
OWL group	0.16	0.03	-0.05	-0.05	-0.01	-0.02	-0.13	-0.10	0.05	-0.03
	(0.49)	(0.08)	(0.06)	(0.42)	(0.07)	(0.05)	(0.13)	(0.20)	(0.05)	(0.05)
HC group	0.60*	-0.03	-0.05	0.94**	-0.14*	-0.08	0.01	0.14	0.00	-0.09*
	(0.31)	(0.05)	(0.03)	(0.37)	(0.08)	(0.06)	(0.13)	(0.16)	(0.04)	(0.05)
Mean at baseline - OWL group	8.90	0.86	0.15	9.34	0.84	0.10	-1.33	-0.94	0.29	0.24
	(1.88)	(0.34)	(0.36)	(1.74)	(0.37)	(0.30)	(1.55)	(1.77)	(0.46)	(0.43)
Mean at baseline - HC group	8.81	0.87	0.14	8.84	0.89	0.14	-1.66	-1.18	0.38	0.30
	(1.74)	(0.34)	(0.35)	(1.76)	(0.32)	(0.35)	(1.57)	(1.80)	(0.49)	(0.46)
Mean at baseline – control group	9.14	0.89	0.09	9.38	0.86	0.07	-1.56	-0.94	0.39	0.24
	(1.69)	(0.32)	(0.29)	(1.71)	(0.35)	(0.25)	(1.71)	(1.81)	(0.49)	(0.43)
Number of observations (N)	940	940	940	385	385	385	902	877	902	877
p-value	0.17	0.72	0.27	0.03	0.17	0.42	0.50	0.41	0.65	0.22

Notes: Treatment groups are older women leaders (OWL) and health committee members (HC). Comparison is to a control group that did not receive any program services. All estimates control for child age and sex, clustering and attrition. Values are coefficients (SE) or mean (SD). * $p < 0.10$; ** $p < 0.05$; *** $p < 0.01$.
